# Buffalo Medical and Surgical Association

**Published:** 1890-10

**Authors:** W. H. Bergtold

**Affiliations:** Secretary


### Society Proceedings.

### BUFFALO MEDICAL AND SURGICAL ASSOCIATION.

Reported by W. H. BERGTOLD, Secretary.

REGULAR monthly meeting was held in the Parlors of the Hotel Iroquois, on September 2d, at 8.15 P. M., with the president, Dr. A. A. Hubbell, in the chair.

Dr. JOHN M. GOLTRA and M. A. Crockett were proposed for membership.

### PUERPERAL FEVER.

BY S. G. DORR, M. D., Buffalo.

There will be no effort made in this paper to run through the etiology, morbid anatomy, or mathematical percentage of the different subjects presented. Of all these things you have a well-founded, general knowledge.

There are but a few questions which I intend to present for your consideration this evening :

1. Is puerperal fever epidemic ?
2. Does puerperal fever depend on a special germ which is pathognomonic ?
3. Is it auto-genetic or self-infectious ?
4. Does infection take place previous to labor ?
5. Treatment.

1. *Is puerperal fever epidemic ?* — The technical word epidemic as applied to disease, has several different meanings attached to it, all of which include the notion of general prevalence among a people or community. Some physicians, as well as writers, make contagion the essence of epidemicity. Etymology does not justify such a conception of the term epidemic. The best writers use the word as signifying wide-spread causes, such as atmospheric, acting at the same moment of time on many individuals, or as something regarding which speculation is vain and is referred to as an epidemic influence. The promiscuous use of the word epidemic in medical literature and medical talk is to be deplored ; to the mind of the medical student it produces hopeless confusion.

There is plenty of testimony showing a connection between puerperal fever and the zymotic diseases, such as scarlatina, diphtheria, erysipelas, etc., but curiously there is an entire absence of testimony showing puerperal fever to be any more abundant during the prevalence of these diseases than at other times. The season of the year having more influence than any other factor outside of hospital infection, twice as many cases occur in the six months from December to May, as from June to November ; and the smallest number of cases occurring during the year being in the months of September and October. This is not to be accounted for, however, by epidemic influence, but by the more constant contact of septic material during the Winter months. The outside air during cold weather is far more pure, but the inside air, the clothing, the bed and the house are more impure.

That deleterious material may find other channels for entering the system than a wounded surface, is evident. French writers report instances of toxemic conditions developing in young midwives during puerperal fever epidemics. When reading such reports I cannot feel safe in following the writer, for I feel he has mistaken frequently a cause for a result.

The point upon which I wish to dwell is that it *is* possible to trace epidemics of puerperal fever directly to the carrying of puerperal poisons from patient to patient through the media of attendants, instruments, and surroundings.

In theory, if not in practice, the doctrine of contagiousness has ceased to be a subject of dispute, and the epidemic idea is little ventured by the well-informed.

The majority of all medical literature at this time declares this fever to be an infectious disease, due to some form of septic inoculation of the genital tract ; an infection in *point of fact* which might be operative in the *non-puerperal* condition, and which is developed and increased in activity or virulence by the parturient. The hypothesis for this increased activity or virulence is the fact that in the capillary network of the blood-vessels of the uterus a great difference exists between the puerperal and the non-puerperal organ.

In the non-puerperal uterus the blood current moves much more rapidly through the capillary blood-vessels than it does through the distended capillaries of the puerperal uterus.

It has been frequently demonstrated that when the micrococci come in contact with the red blood corpuscles in a state of rest or of but little motion, they — the red blood corpuscles — will stick together, forming thrombi and micrococci, very rapidly increase in numbers, whereas, in a blood current, the opposite condition prevails, (viz.:) no thrombi, no increase of micrococci. I am of those who believe there is no such thing as puerperal fever as a distinct disease, standing independent and alone. I believe it to be really a surgical fever, modified by the particular septic germs in conjunction with the physiological conditions which belong to the puerperal state, such as blood changes induced by pregnancy ; the deep situation of puerperal wounds ; the presence of clots even in the capillary net-work of the uterus ; disintegrating and decomposing tissue; the ease with which lymphatic absorption takes place, the size of the veins already referred to, and the proximity of the peritoneal cavity.

2. *Does it depend on a special germ which is pathognomonic ?* —Up to the present time no distinct specific bacterial form has been demonstrated (that I am aware of) peculiar to puerperal fever only, the different, though closely allied bacterium of pyemia, septicemia, gangrene, erysipelas and diphtheria, all having a common connecting link in being generated in putrefying media, seem to have the power of producing puerperal fever. Curiously, enough, the bacterium termo and bacterium commune, to which the putrefaction is largely due, are in themselves harmless, except it may be to produce putrid intoxication. We know clinically that we may have septicemia without pus, and know we may have abundant pus and no septicemia.

It has been demonstrated that the supposed harmless spores found on leaves, fruit, and even in supposed pure air, can by a succession of cultures be brought to flourish in a warm alkaline fluid, and that they acquire a capacity to penetrate living tissues, to proliferate in them, to excite necrosis and to cause death.

Doderlein, Van Ott and Winter, three eminent German bacteriologists, have, independently, examined the lochia of healthy lying-in-women to ascertain the presence of microbes. They all agree in their results (viz.:), that the lochia contained in the uterus are free from germs, while the discharges in the vagina contain abundance of germs of many different kinds. They also examined the secretions of the genital canal in the unimpregnated state with corresponding result (viz.:), the secretions from the tubes and the uterus were free from microbes, those from the vagina contained germs in great numbers and variety. These results harmonize with our clinical experience. They explain why a retained fetus and secundines in utero do not putrefy, while the same products, if detained in vagina, quickly decompose.

All of you must have been surprised and gratified at times to see that a dead fetus entombed in the uterus for months made no trouble; that when it was expelled it was brown, dry and mummified.

3. *Is it self-infectious ?*— Here comes the question of self-infection; (a term which I prefer to auto-genetic). It is a practical point relative to treatment, and must settle the advisability of removing the contents of the uterus or not, and how is best to do it ?

If there is no danger of self-infection, then it is best to let them alone. If there is danger of self-infection from the vagina, then vaginal antiseptics best be applied.

The uterine cavity of lying-in-women, where there was febrile symptoms, have been found to contain streptococci, and in women dying from septicemia, not only the uterus but every organ of the body contained them. Self-infection without a cause, I do not believe in, but a self-infection without contact of hands or instruments, I do believe may come from the vagina and other surroundings. I also believe that deleterious material *may* find other channels for entering the system than a wounded surface ; but I am in doubt if there be cases of self-infection which result from processes within the patient herself, quite independent of the entry of germs, as is claimed by Dr. Barnes.

4. *Does infection take place previous to labor ?*— The question of infection previous to labor is very obscure. It is important to know if it exists, and if such a condition does exist, then it is important to know what the noxious material may be and how it affects the patient.

I hope the members present, in their discussion, will be able to afford much light on the subject. It has been my fortune to have had several cases where a fever existed at the time or immediately subsequent to labor. I have no record of them only in memory. These cases have been frequently difficult, chronic and dangerous ; more so, I think, than those where the infection occurred subsequent to labor.

It will be easily apprehended that it may arise in any period of pregnancy or of the puerperal state. It seems to me to be a metritis or a parametritis, more than a phlebitis in the beginning ; although these cases may result in a subsequent phlegmasia albo-dolens, they, in my experience, more frequently result, however, in abscesses or peritonitis. We not frequently see the original inflammatory action of the womb and its immediate surroundings disappear, while the more remote parts of the body may be suffering acutely.

My experience has led me to believe that a septic infection does take place previous to labor, and that it is a very different disease from that which occurs subsequent to labor. It is more chronic in all of its characteristics and more likely to form abscesses.

5. *Treatment.*— The present unanimity of treatment of this disease is curious. Most articles on the subject seem to wind up with curetting the uterus and bichloride irrigations. This monotony of treatment is broken, however, by differences of opinion relative to the percentage of the bichloride.

The young man must feel as though he had not discharged his duty to his patient, unless he has scraped the uterine cavity and irrigated the same with bichloride. I do not criticise the curetting of the uterus when it is necessary, but I do not think it is so very often necessary, and that frequently it is harmful rather than advantageous.

In regard to bichloride of mercury irrigations, I must say, I have been unfortunate in not having seen any remarkable good results from their use. I do not criticise the cleansing of the uterine cavity, but I think we have a better agent in iodine.

Iodine may not be the most powerful germicide, but its wide range of curative powers, in removing inflammations, healing sores and alterative action, makes it the better agent.

Bichloride of mercury is undoubtedly a good antiseptic for hands and instruments; they will be less colored by it than by iodine, — then again, the hands and instruments differ widely from the diseased parts of the patient.

Pepper, in 1886, recommended the introducing of iodoform suppositories into the uterine cavity, which treatment I have tried with varying success. Sometimes the patient did better when the treatment was stopped; in fact, I have felt at times that the recovery of the patient was due to its suspension. That which has given me the most satisfaction for several years, has been the simple vaginal wash of compound tincture of iodine, one drachm to the pint of warm water — irrigation repeated every six to eight hours. Upon this simple wash I have relied successfully in those very bad cases of the disease which frequently appear in that locality where my lot is cast.

Treatment is determined in great measure by the tissue system which is predominately affected. There may be inflammation of the mucous membrane of the genitals only, or there may be inflammation of the uterine parenchyma, or inflammation of the peritoneum, covering the uterus and contiguous parts, or a phlebitis with pyemia or a septicemia, or an absorption of ptomaines with toxic affects,—all these conditions are to be met with good judgment.

The fear of carrying the disease from patient to patient is great, but I now think quite unnecessary. When we feel that we are not common carriers in this respect, we are more brave. When we can show that the dirt, dust, filth, blood, heat, etc., will produce the disease, when taken in connection with the bacterium of every unwashed vagina, then you cannot fear to point out the true cause of the trouble to the patient and friends. So far as preventive medicine is concerned, I am convinced that antiseptic vaginal irrigations are sufficient.

### Strangulated Hernia.

By Dr. W. S. TREMAINE, Buffalo, N. Y.

(*Abstract.*)

Dr. Tremaine said that he was well aware that he had not chosen a very new subject, but he also knew that it was not worn so thread-bare but that it might afford material for some practical points for the general practitioner. Twenty or thirty years ago our teachers said that in cases of strangulated hernia, we were to continue our efforts at reduction for a long time, and avoid, if possible, an operation. There remains with the older men, more or less, of the traces of this early teaching, and he has occasion as a consultant to meet with it now and then. The first point of his remarks was directed to the absolute necessity of early operation in cases of strangulation, if gentle taxis failed to reduce. No case of strangulated hernia will die from an operation, if in proper hands; but will surely die if let alone, and especially if too great and vigorous efforts at reduction are used. Every man who practises when he is not within easy reach of a competent surgeon, should know how to operate for strangulated hernia. A practitioner on encountering a case of strangulated hernia, should either send at once for a consultant, or explain the risks of an operation and the necessity for one. Then, if under chloroform a reduction with slight and exceedingly gentle taxis cannot be secured, the hernia is to be attacked, the sac opened, and the construction found and released. He related a case where, from the severe and prolonged efforts at taxis, he found the gut black, and he hesitated whether to return it to the peritoneal cavity, or make an artificial anus. He remembered Sir Astley Cooper's axiom: "That the belly is the best place for a gangrenous gut," and slipped it back. The case made a good recovery. Dr. Tremaine's second point was in relation to the so-called "radical cures of hernia," which he believes ought to be termed "attempted radical cures." We are as yet a long way from being at the bottom of this matter. There is at present a sort of rage for operating on cases of hernia, mostly because of the simplicity of the operation, and the comparatively small risks. In cases with small sacs, little gut, or perhaps mostly omentum, he would not operate. There is now a current setting in the other direction, and a re-action against this promiscuous operating is taking place. He mentioned a case where it was exceedingly difficult to isolate the sac, the hernia was omental, and had probably never been entirely reduced; he found the cord, the testicle, the omentum, and the sac, one intricate, tangled mass, and was compelled to remove the whole. It was a question of moment, in this case, whether he ought to the have removed testicle without the patient's consent; all of the attending circumstances certainly justified his decision to do so. There is no fixed point at present from which we can decide whether we ought to attempt a radical cure or not. Dr. Tremaine believes that, if the patient be a person who works by his brain, and not by arduous labor, and whose rupture is easily retained by a simple truss, that such a case should be left unoperated. If, however, the patient be one who does great muscular labor, and who cannot maintain his hernia within the abdomen easily by a truss, then in such a case we should attempt a radical cure. In cases of strangulation, and irreducible cases, it is a question yet unsettled, whether, when operating for relief, we should attempt to get a radical cure also. In these last cases deaths do occur, and we cannot always say from what cause, either.

The patient's life is always to be the first consideration, and whatever arises, the consultant's judgment ought to prevail.

DISCUSSION.

Dr. M. D. Mann thinks that Dr. Dorr's views are, in the main, accepted by nearly every one. Germs, from a case of puerperal fever, are more virulent than similar germs from another soil, as, for example, the normal vagina.

It is not necessary that a man should give up all obstetric work, because he has a puerperal fever case arise in his practice ; he must, of course, exercise every precaution possible. He has frequently gone directly from such a case to a laparatomy without doing harm. Of course, it is understood that he changed his clothes, took a thorough bath, and bathed the hands and beard with an antiseptic solution before meeting the operative patient.

Dr. Frederick said that there was not much left for discussion, as Dr. Dorr's paper had covered the ground very thoroughly in a manner acceptable to nearly all. He believed that the essayist had not laid stress enough on the efficacy, in proper cases, of intrauterine douches.

Vaginal douching is enough if the lesions are in the vagina or about the cervix, but they will fail utterly to do good if the trouble be in the uterus proper.

Dr. F. W. Bartlett said that he had lost very few cases of a puerperal nature, and it seemed to him that those which he did lose died after having used intrauterine douches. One case had a chill directly after the exhibition of an intrauterine douche, and died directly after the second douche was given. In another case the patient died twelve hours after the second douche was used. In some cases of pre-puerperal fever it has seemed to him as though there was a typhoid condition present. In all events, we ought always to be thoroughly antiseptic. He often uses a finger-cot in making examinations.

Dr. Wyckoff thinks that we are nearly all agreed as to the nature of puerperal fever, but believes that intrauterine douches may be very dangerous.

Dr. Ingraham said that he believes most thoroughly in the use of the curette, and intrauterine douche when necessary. He has never seen any trouble arise from their use.

Dr. Persons thought that Dr. Dorr's ideas had a tendency to combat the theories now taught in the schools. The first thing to do in a case of puerperal trouble is to locate the cause, and then treat it. He uses a speculum for this purpose, and he now has much less difficulty than formerly in locating the offending lesion. If it be in the vagina, or about the cervix, vaginal douches are enough, but if in the uterus, uterine douches are positively indicated.

Dr. Coakley said that in thirty years' practice he has never had a well-marked case of puerperal fever. He has always been exceedingly careful, and has practiced scrupulous cleanliness. He uses turpentine as a hand-wash before making vaginal examinations.

Dr. Dorr said that he was sorry that the discussion had not been more on those obscure cases of pre-puerperal fever, and he hoped that those present would give their experience with such cases.

Dr. Mann recalls, in particular, two cases of dead fetus where there was fever previous to the labor ; here it was easy to explain the fever. In other cases he has seen fever before parturition, and in many could not explain it at all.

Dr. Phelps agrees entirely with Dr. Tremaine as to the necessity of having every practitioner know how to operate for strangulated hernia. Operations for hernia are a bugbear to nearly every student and practitioner. The late Dr. J. F. Miner said that a hernia really had but two coverings : The skin and the sac, instead of seven as given in the text-books ; we ought to, therefore, cut down, carefully of course, but boldly, until the gut is reached, and then liberate it. Antisepsis in these operations is of the utmost importance and should be exercised in every necessary detail. He does not believe in taxis, except of the gentlest kind, and continued but a short time. He believes that omentum which has been squeezed for a long time by efforts at reduction ought to be cut off, etc. Dr. Phelps also believes that we ought to try to get a radical cure in operations undertaken for the relief of strangulated cases. In old people and large hernias we ought, however, to simply try for relief, and not for radical cure. Children and young people bear it well. We can't say why one case is cured, and a second not. The death-rate is very small. He does not recommend operative procedures in children before seven years old.

Dr. Bartlett asked about the injection methods of cure for hernia. In trusses for infants with congenital umbilical hernia, he believes that the hemispherical button thereon is more apt to keep the breech open by pressure than to lead to its closure.

Dr. Tremaine said that he believes the mortality in operations in non-strangulated cases of hernia is about three per cent. In cases where the rupture is small, and easily held in place by a truss, he always hesitates to operate. He does not mean to decry the efforts to cure hernia by operation, but he wishes to impress the need for caution in entering upon an operation. He mentioned a case in a sailor, where he had used chromated cat-gut to unite the pillars of the ring, and after five years there was no return. This is his only case where he thinks he is justified in saying that a radical cure was obtained. The injection method has been tried and found wanting. He spoke of the use of electricity, now practiced by a notorious quack of Buffalo, for the cure of hernia; he thinks that it acts by exciting adhesive changes about the ring, and he mentioned a case where he had to operate for strangulation: The patient had been pronounced cured, after treatment by this quack, and a few days afterwards was taken with symptoms of strangulation. On opening the sac he found that the stoppage had been incomplete; that the gut was patulous above and below, but that it was firmly bound to the ring by fibrous adhesions, which had partly constricted the lumen of the gut; the constricted portion itself was gangrenous.

Under voluntary communications, Dr. Ingraham spoke of a visit which he had made to the gynecological institute of Dr. Leopold, of Dresden, and described the method of work, etc.

Dr. C. G. Stockton spoke of his experience in attendance at some of the medical society meetings in London.

It was announced that Dr. Edward Clark would read a paper on Abscess and Fistula of the Ano-rectal Region at the October meeting.

